# Development and Validation of Sentences Without Semantic Context to Complement the Basic English Lexicon Sentences

**DOI:** 10.1044/2020_JSLHR-20-00174

**Published:** 2020-10-13

**Authors:** Erin R. O'Neill, Morgan N. Parke, Heather A. Kreft, Andrew J. Oxenham

**Affiliations:** a Department of Psychology, University of Minnesota, Minneapolis

## Abstract

**Purpose:**

The goal of this study was to develop and validate a new corpus of sentences without semantic context to facilitate research aimed at isolating the effects of semantic context in speech perception.

**Method:**

The newly developed corpus contains nonsensical sentences but is matched in vocabulary and syntactic structure to the existing Basic English Lexicon (BEL) corpus. It consists of 20 lists, with each list containing 25 sentences and each sentence having four keywords. Each new list contains the same keywords as the respective list in the original BEL corpus, but the keywords within each list are scrambled across sentences to eliminate semantic context within each sentence, while maintaining the original syntactic structure. All sentences in the original and nonsense BEL corpora were recorded by the same two male and two female talkers.

**Results:**

Mean intelligibility scores for each list were estimated by calculating the mean proportion of correct keywords achieved by 40 normal-hearing listeners for one male and one female talker. Although small but significant differences were found between some pairs of lists, mean performance for all 20 lists fell within the 95% confidence intervals of the mean.

**Conclusions:**

Lists in the newly developed nonsense corpus are reasonably well equated for difficulty and can be used interchangeably in a randomized experimental design. Both the original and nonsense BEL sentences, all recorded by the same four talkers, are publicly available.

**Supplemental Material:**

https://doi.org/10.23641/asha.13022900

A number of different materials have been used in clinical and research settings to assess listeners' ability to understand speech. These include sentence lists, such as the Hearing in Noise Test (Nilsson et al., 1994), AzBio sentences (Spahr et al., 2012), the Bamford–Kowal–Bench Speech-in-Noise Test (Bench et al., 1979), and the Quick Speech-in-Noise Test (Killion et al., 2004), as well as isolated words, such as the consonant-nucleus-consonant (CNC) word list (Nilsson et al., 1996) and spondees (e.g., Harris, 1991; Turner et al., 2004). Sentences have some advantages over isolated words, in that they incorporate the co-articulation between words that occurs in natural communication. They also include varying degrees of semantic context that makes many words in sentences predictable to some extent, based on the preceding or following words. Although the inclusion of semantic context has ecological validity, it also imposes a degree of uncertainty regarding what was actually heard, as opposed to simply inferred, by the participants. This process of inferring, or “filling in,” may inflate the estimated audibility of speech and may potentially discount any additional listening effort exerted to achieve a given level of speech understanding (e.g., Sarampalis et al., 2009; Winn, 2016).

Interest in listening effort has grown in recent years in response to individuals with hearing loss and cochlear implants (CIs) reporting high levels of mental fatigue associated with daily listening (Hughes et al., 2018). Increased listening effort has also been inferred from laboratory studies using measures such as pupil dilation (e.g., Beatty, 1982; Kahneman & Beatty, 1966), which may result at least in part from increased semantic inference or “filling in” on the part of CI users (Winn, 2016). The Framework for Understanding Effortful Listening, developed by Pichora-Fuller et al. (2016), highlights the interaction between the semantic context and vocabulary of a speech passage and the differential cognitive and listening effort required to decode such passages. Other studies have also explored the difference in the use of semantic context between children and adults, with some indicating that semantic context is only maximally leveraged in adulthood and, as a result, children need higher levels of audibility to achieve the same level of speech understanding (Nittrouer & Boothroyd, 1990; Stelmachowicz et al., 2000).

Despite the growing interest in the connections between the use of semantic context, listening effort, development, and hearing loss, the speech materials currently available to explore this issue in adults remain limited. Most commonly, a set of sentences is used for which the final word in each sentence is either predictable or not, based on the preceding words in the sentence (Bilger et al., 1984; Lash et al., 2013). This type of sentence provides a controlled method for analyzing the effect of semantic context, but places artificial importance on the final word of each sentence that is not directly comparable to the ways in which semantic context operates in conversational speech typical of everyday environments. For example, listeners may use context occurring later in a sentence to resolve earlier ambiguities. In addition, the resulting measure of speech perception is limited to just one word per sentence, making it a relatively inefficient method to estimate overall intelligibility.

Some studies have tackled this problem from a different angle, using stimuli that eliminate semantic context by scrambling all the words in a sentence, but violate typical grammatical structure in the process (Boothroyd & Nittrouer, 1988). Other studies have maintained correct grammatical structure but have removed semantic context by replacing nouns and verbs with novel tokens, such as pseudowords (Carroll, 1883; Yamada & Neville, 2007). Many studies also utilize matrix sentences that maintain a consistent sentence structure (e.g., proper name + verb + number + color + noun; e.g., Bolia et al., 2000; Hagerman, 1982; Kollmeier et al., 2015), but the closed-set nature of the measure and limited number of choices for each word category make the results hard to interpret in terms of everyday speech perception outcomes. A perhaps more ecologically valid nonsense sentence corpus has been used to examine audio-visual cues on speech perception (Helfer, 1997) and to study the intelligibility of whispered speech (Freyman et al., 2012; Ruggles et al., 2014). It contains sentences with English words, typical grammar, syntax, and prosody, but no semantic context. These sentences thus sound correct, but do not make any logical sense. These “nonsense sentences” provide more information than sentences with only one target word and arguably provide more ecological validity than sentences with invalid syntax or sentences selected from a closed and known set of words in each position.

Although a nonsense sentence corpus offers the opportunity to study sentence perception in the absence of semantic context, it is difficult to quantify the effect of such semantic context because there is currently no equated sentence corpus containing semantic context for comparison. Specifically, if performance on these nonsense sentences is compared to performance on another existing corpus with semantic context, such as the AzBio (Spahr et al., 2012) or IEEE (1969) sentences, the influence of different talkers, vocabulary, number of keywords, and sentence length inherent to the different corpora cannot be eliminated as confounds (O'Neill et al., 2019). Although Boothroyd and Nittrouer (1988) and Stelmachowicz et al. (2000) constructed sentences with low and high semantic context, the vocabularly was targeted at young children, and the number of sentences was small compared to those of other commonly used corpora.

The purpose of this study was to develop, record, and validate a new nonsense sentence corpus, matched to an existing sentence corpus in terms of talkers, vocabulary, number of keywords, and sentence length. The Basic English Lexicon (BEL) sentence corpus (Calandruccio & Smiljanic, 2012) was employed due to its simple vocabulary and sentence structure, as well as its high degree of semantic predictability. The BEL sentences were originally designed to probe the hearing status of nonnative English speakers who may have limited knowledge of complex English vocabulary. The keywords in the corpus were derived from interviews with 100 nonnative English speakers, which focused on 20 topic categories, such as cooking, sports, extended family, work, and others. The keywords were then distributed across 20 lists of 25 sentences each, balancing the rate of occurrence of each keyword, syntactic structure of sentences, syllable counts, and high-frequency speech information (i.e., fricatives, affricatives). Native English speakers then listened to the sentences in background noise to ensure equal perceptual difficulty across lists. The new nonsense sentence corpus was developed in such a way that the keywords within each list were maintained from the original corpus, along with the grammatical structure of each sentence. Keywords within each list were scrambled across sentences, so the final lists consisted of sentences with typical grammatical structure but without semantic context. The resulting sentences were therefore syntactically correct and fully formed English sentences, but were also extremely unlikely and unpredictable contextually. Both the original and new nonsense BEL sentences were then recorded by the same two male and two female talkers (one younger and one older in each pair). Finally, normal-hearing participants were tested on the nonsense sentences spoken by one of the female and one of the male talkers to ensure that the keywords were not predictable and that the lists were balanced for intelligibility by normal-hearing listeners in the presence of background noise.

## Method

### Sentence Development

To maintain lexical and grammatical consistency with the original BEL sentence corpus, all lists in the nonsense BEL corpus contained the same vocabulary, keywords, and sentence structures as the original 20 lists of 25 sentences each. The original BEL sentence corpus utilizes different variations of a basic syntactic framework that consists of combinations of the following word categories: determiner (D), adjective (A), noun (N), pronoun (Pro), adverb (Adv), verb (V), and preposition (P). Although a number of different combinations of these basic word categories were used to create various syntactic structures, 12 variants account for 70% of all syntactic frameworks, with the remaining 30% of variants being slightly less common but still basic in the sense that they lacked syntactic complexity, such as embedded or proposed dependent clauses. An example of one of the common syntactic structures used is DANVA, with a corresponding sentence being, “*The boiled fish smells bad*.” To ensure that all sentence structures in the nonsense corpus matched those used in the original BEL corpus, the original syntactic structure for every sentence was identified, and each word was sorted into its appropriate word category. Once all of the words from sentences within a list were sorted into word categories, these words were randomized by computer within each word category and added back into the original sentence structures to create novel nonsense sentences. The randomly assigned combination of words from appropriate word categories (as determined by the necessary syntactic structure of the sentence) were then checked manually for verb tense and noun plurality agreement, and if any disagreement was found, the word causing the grammatical violation was replaced with another randomly selected word from that word category, until the grammatical features were in agreement. The resulting 20 novel lists contained the same 25 sentence structures and 100 keywords as the original BEL sentence list from which it was derived, but was devoid of semantic context. An example of one such nonsense list is shown in Table 1. The complete list of all 500 nonsense sentences can be found in the Supplemental Material S1.

**Table 1.** List 11 of the original Basic English Lexicon corpus and Basic English Lexicon nonsense corpus.

Syntactic structure	Original sentence using described structure	Nonsense sentence using same structure
DNVPAN	The **MEETING STARTS** in **TWENTY MINUTES**.	The WINDOWS LEARNED in BROWN SECRETARY.
DNVAN	The CUSTOMERS HATE BLACK TEA.	The MEAL PLANNED **TWENTY** KIDS.
DANVA	The SICK PERSON FEELS BETTER.	That COOL ROOM DRINKS HERE.
DANVAdvA	That BROWN BIRD is ALWAYS HERE.	A DANGEROUS BIRD was REALLY ORANGE.
DANVProAN	The THREE COUSINS did their MATH HOMEWORK.	The TWO GLOVES had their ENGLISH HORSE.
DANVDN	The DARK CLOUD COVERED the SKY.	The CHICKEN MOVIE CLIMBED the SON.
DANVN	The GROCERY STORE SELLS FOOD.	The GROCERY PERSON NEEDS FARM.
DNVPDAN	The MOVIE STARTED in the SMALL ROOM.	The CAKE BIT in the BETTER SOUP.
DANVDAN	The CHICKEN SOUP was a TASTY MEAL.	The DIFFICULT JUICE was the BIRTHDAY TEST.
DANVAandA	The COOL NIGHT was COMFORTABLE and RELAXING.	The TASTY NIGHT was THREE and DARK.
DANVVAdv	The BIRTHDAY CARD was SENT LATE.	The TROUBLED GRADE is SENT EASILY.
DNVNAdv	The SECRETARY LEARNED SPANISH EASILY.	A RADIO FEELS PROFESSOR LOUDLY.
DANVPDN	The WHITE HORSE LIVES on a FARM.	The SMALL TEA SELLS over an APARTMENT.
ProNVAN	Our APARTMENT NEEDS MORE WINDOWS.	Our HOMEWORK BUYS MORE SKY.
ProNVAN	Our MOTHER DRINKS ORANGE JUICE.	Our RABBIT STARTED SICK CUSTOMERS.
DANVDN	The DANGEROUS SNAKE BIT the RABBIT.	A COMFORTABLE BOYFRIEND COVERED theCOUSINS.
DNVDAN	The PROFESSOR GAVE an UNFAIR GRADE.	The SNAKE PLAYED the UNFAIR WEDDING.
ProVANPDN	They PLAYED FAST MUSIC on the RADIO.	They HATE GREEN **MINUTES** on the CARD.
DANVAdvA	That ENGLISH TEST was REALLY DIFFICULT.	The MATH STORE was ALWAYS RELAXING.
DNandNVProN	The BOYFRIEND and GIRLFRIEND PLANNED their WEDDING.	The SISTER and CLOUD SCREAMED their MOTHER.
DNVAdvPDN	The KIDS SCREAMED LOUDLY in the PARK.	The **MEETING** LIVES LATE on the FENCE.
DANVN	The TROUBLED SON STOLE MONEY.	The WHITE MONEY GAVE KITTEN.
ProNVNAdv	His SISTER BUYS CAKE DAILY.	His SNOWMAN STOLE SPANISH DAILY.
DANVPDN	A LITTLE KITTEN CLIMBED over the FENCE.	The LITTLE GIRLFRIEND **STARTS** in that MUSIC.
DNVAAN	The SNOWMAN had TWO GREEN GLOVES.	The PARK did FAST BLACK FOOD.

*Note.* The syntactic structure for each sentence is shown in the far left column with word categories as follows: D = determiner, A = adjective, N = noun, V = verb, P = preposition, Adv = adverb, Pro = pronoun. Keywords are in uppercase and the four bold words indicate an example of how words were redistributed from one original sentence across the nonsense sentences.

### Sentence Recordings

Four native speakers of American English, two women (aged 20 and 62 years) and two men (aged 26 and 63 years), recorded all 500 original BEL sentences, as well as all 500 newly created nonsense BEL sentences. All sentences were digitally recorded in a single-walled, sound-attenuating booth located in a quiet room, at a 22050-Hz sampling rate with 16-bit resolution, using a PMD670 solid state recorder (Marantz). Talkers were seated approximately 12 in. from an ME64 stationary microphone (Sennheiser) and instructed to keep their backs against the back of the chair to maintain a roughly constant distance from the microphone. Talkers were also instructed to maintain a stable level of speech and to read each sentence in a natural conversational manner. Each list of 25 sentences was printed on a separate sheet of paper, and talkers were instructed to pause in between lists, to avoid any sound contamination of the sentences due to page turns. Talkers were also instructed to take slightly longer than natural pauses in between each sentence, to aid with the subsequent splicing process. Finally, if the talkers hesitated or made a noticeable error, they were instructed to pause and repeat the sentence.

After the initial recording session by each talker, the sound files were digitally edited using Audacity software (free audio editor, Version 2.3.3, 2019) and each sentence was spliced and saved as an individual sound file. Each audio file was then cross-checked with the text of each sentence to ensure word accuracy, and was also checked for any audible distortions, microphone pops, clipping, or ambient sound. After the initial editing, each talker rerecorded any flagged sentences and the editing process was repeated until all sentence recordings were deemed adequate. A two-way repeated-measures analysis of variance (with a Huyhn–Feldt correction for lack of sphericity), with average sentence duration per list as a dependent variable and talker and corpus as factors, showed a significant effect of corpus (original vs. nonsense), *F*(1, 19) = 196.8, *p* < .001, η_p_^2^ = .912, and talker, *F*(2.5, 47.9) = 234.9, *p* < .001, η_p_^2^ = .925, and a significant interaction between corpus and talker, *F*(2, 38.9) = 7.8, *p* = .001, η_p_^2^ = .291. On average, sentence durations were longer in the nonsense corpus (2.47 s) than in the original corpus (2.27 s), but each talker also spoke at different rates, which impacted overall durations for each corpus differently. The fastest talker was the older male (mean sentence duration = 2.16 s), followed by the younger female (2.27 s) and the younger male (2.37 s), with the older female having the slowest average speaking rate (2.67 s). Recordings from all four talkers for both the original BEL corpus and the nonsense BEL corpus are available for download from the Data Repository for the University of Minnesota (DRUM) at https://doi.org/10.13020/tdf8-6p56.

### Evaluation of Predictability

To ensure the original BEL corpus and nonsense BEL corpus did indeed differ in predictability due to semantic context, a “fill-in-the-blank” test was administered to 20 native speakers of American English. The participants (14 female, six male) were mostly undergraduate students and ranged in age from 19 to 30 years (*M* = 20.9 years, *SD* = 2.7). All experimental protocols were approved by the institutional review board of the University of Minnesota, and all participants provided informed written consent prior to enrollment. For each sentence in the original and nonsense BEL corpora, one keyword was randomly omitted and replaced by a blank. Sentences from two original lists and two nonsense lists were then combined in random order to create a 100-sentence fill-in-the-blank test for each participant. This was done for all lists of sentences so that each participant completed four lists and each of the 40 lists was completed by two participants. An excerpt from one of the fill-in-the-blank tests is shown in Table 2.

**Table 2.** Example sentences from the fill in the blank test.

Sentences shown to participants	Missing keyword	Sentence type
1. The hungry teenagers eat _______.	snacks	Original
2. A shy _______ traveled the people.	cousin	Nonsense
3. The woman was weird in _______ problems.	many	Nonsense
4. The _______ band played in a concert.	popular	Original
5. The milk and cheese smelled _______.	horrible	Original
6. The thirsty _______ was excited and black.	dish	Nonsense
7. The people write after her _______.	salad	Nonsense
8. Her neighbors are _______ and not silver.	bright	Nonsense
9. The bears eat brown _______.	performer	Nonsense
10. The class broke _______ twins.	scary	Nonsense

*Note.* The left column shows how the sentences appeared on the fill in the blank test. The middle column shows the missing word from each sentence, and the far right column denoted the corpus to which each sentence belongs.

The tests were administered on a computer in a quiet room, with participants typing their answers into a PDF document with an empty cell corresponding to each blank. Participants were instructed to fill in the blank in each sentence with the word they thought would fit best. Participants were told to type only one word per blank and to guess when they were unsure of the correct answer. Responses were scored in three ways. For the first scoring method, a response was only considered to be correct if it exactly matched the actual missing keyword. Since the correct verb tense was often ambiguous, the second scoring method also considered responses to be correct if the verb was correctly identified but the tense was incorrect. For example, if the missing keyword was “drinks,” but a participant answered “drank,” that response would be considered correct. In the final scoring method, any response that was a synonym, an antonym, or in the same semantic category as the actual missing keyword was considered correct. For example, if the missing keyword was “store,” but a participant responded with the word “shop,” the response would be marked as correct. A list of the original words along with the words that were accepted as having a similar semantic meaning is provided in the Supplemental Material S2. Taken together, these three scoring methods provided a more nuanced interpretation of the data, especially when considering how ambiguities may have been resolved if the sentences had been spoken, rather than read.

### List Validation

To confirm that the 20 newly created lists of nonsense BEL sentences were equated for difficulty and could be used interchangeably in experimental design, 40 young, normal-hearing adults listened to all 20 lists of nonsense sentences, in background noise. Only three had previously participated in the written validation test described above, which had taken place 6 months prior. Twenty participants (19 women, one man) ranging in age from 18 to 22 years (*M* = 19.5 years, *SD* = 2.2) listened to sentences recorded by the older female talker, and 20 participants (15 women, five men) ranging in age from 18 to 24 years (*M* = 20.9 years, *SD* = 2.7) listened to sentences recorded by the younger male talker. Though traditionally validation studies have been conducted using only one talker (e.g., Calandruccio & Smiljanic, 2012; Nilsson et al., 1994), we chose to include two of the four recorded talkers for a more robust, gender- and age-balanced analysis of possible list-level differences in performance, while staying within reasonable time constraints for data collection. Normal hearing was defined as having pure-tone audiometric thresholds less than 20 dB HL at all octave frequencies between 250 and 8000 Hz with no reported history of hearing disorders. All experimental protocols were approved by the institutional review board of the University of Minnesota, and all participants provided informed written consent prior to enrollment.

All sentences were presented in Gaussian noise, spectrally shaped to match the long-term spectrum of the nonsense BEL corpus as recorded by either the older female talker or the younger male talker. Thus, the speech-shaped noise differed for the two groups of participants, but was mixed with the speech at the same signal-to-noise ratio (SNR) of −4 dB. This SNR was selected based on pilot testing to avoid floor and ceiling effects and to facilitate direct comparisons between lists and talkers. The speech was presented at a root-mean-square level of 65 dB SPL, as measured at the position corresponding to the participant's head, and the noise level was adjusted to produce the desired SNR. The noise was gated on 1 s before the beginning of each sentence and gated off 1 s after the end of each sentence. The stimuli were generated using MATLAB and converted via an E22 24-bit digital-to-analog converter (Lynx Studio Technology) at a sampling rate of 22050 Hz. The sounds were presented in a single-walled, sound-attenuating booth located in a quiet room via an amplifier and a single loudspeaker, placed approximately 1 m from the listener at 0° azimuth.

Participants responded to sentences by typing what they heard on a computer keyboard. They were encouraged to guess individual words, even if they had not heard or understood the entire sentence. Instructions were given orally, and participants were asked if they had any questions about procedures before beginning the task. Sentences were scored for keywords correct as a proportion of the total number of keywords presented. Initial scoring was automatic, with each error then checked manually for potential spelling errors or homophones (e.g., wait and weight), which were marked as correct. The 20 lists of nonsense BEL sentences were ordered randomly and split into four blocks (each containing five lists) for each participant. All testing was completed in one 2-hr session per participant with a short break after completion of the first two blocks.

## Results and Discussion

### Predictability of Nonsense and Original BEL Sentences

**Figure 1.**
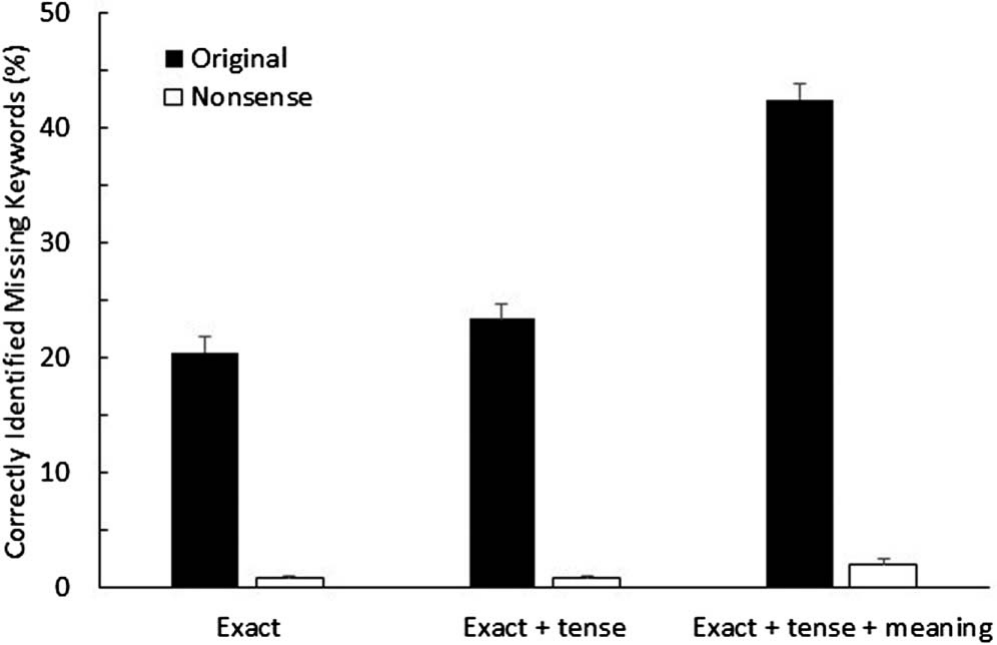
Mean proportion of correctly “filled in” words, averaged across all 20 lists of original Basic English Lexicon (BEL) sentences and nonsense BEL sentences, scored using three methods. The first set of bars represent the mean proportion of reported keywords that were exactly correct (Exact), the second set of bars show the proportion of keywords that were either exactly correctly or contained the right word but wrong tense or plurality (Exact + tense), and the third set of bars show the proportion of reported keywords that were either exactly correct, correct except for an error in tense or plurality, or had the same semantic meaning as the missing keyword (Exact + tense + meaning). Results for the original BEL sentences are shown by black bars and those for the nonsense BEL sentences are shown by white bars. Error bars represent 1 standard error of the mean between listeners.

The mean proportions of correctly “filled in” words for each list of original and nonsense BEL sentences, scored using the three different scoring methods described above, are shown in Figure 1. As expected, participants were able to correctly guess the missing keywords at much higher rates for the original BEL sentences (*M* = 21%) than for the nonsense BEL sentences (*M* < 1%), which was also true when allowing tense or plurality errors (*M* = 22% vs. < 1%). Because of the dichotomous nature of the response variable, a binomial generalized linear mixed-effects model was used to analyze results with the number of correctly guessed keywords (out of the total number of keywords) as the response variable. Fixed effects included material type (original vs. nonsense) and scoring method, while random effects included an intercept for each participant. The model was implemented using the R programming language and the lme4 package (Bates et al., 2015). The significance of the fixed effects was tested by Wald χ^2^ tests in a Type III analysis of deviance. All *p* values were corrected using the Holm correction and compared against a criterion of θ = 0.05 to assess statistical significance. The analysis of deviance confirmed that performance for original versus nonsense sentences was significantly different from each other across all three scoring methods, χ^2^(1) = 91.7, *p* < .001. Post hoc contrast tests showed that this difference was significant when scoring for exact keywords, estimated odds ratio = 2.64, χ^2^(1) = 91.7, *p* < .001, and when allowing tense or plurality errors, estimated odds ratio = 2.65, χ^2^(1) = 101.3, *p* < .001. A significant difference was also found for the rates that counted semantically similar words as correct, 43% and 2% for original and nonsense BEL sentences, respectively; estimated odds ratio = 2.64, χ^2^(1) = 240.6, *p* < .001. These results, showing correct response rates of 2% or less in all cases, confirm that the new nonsense BEL sentences provide minimal semantic context.

### Speech Intelligibility in Noise of Nonsense BEL Sentences

**Figure 2.**
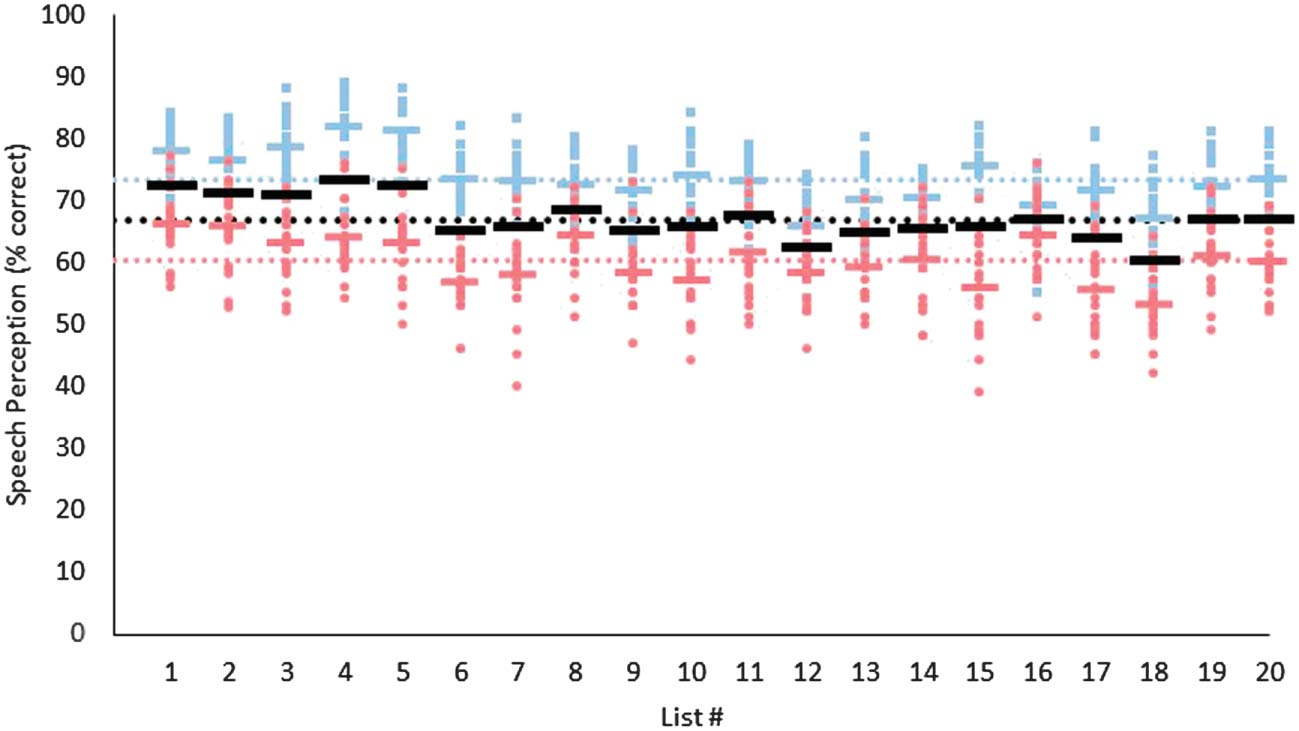
Speech perception for nonsense Basic English Lexicon sentences recorded by an older female talker and a younger male talker. Red circles and blue squares show individual data for the male and female talker, respectively. The red and blue bars indicate mean performance for each list for the male and female talkers, and black bars show list averages across talker. The horizontal red, blue, and black dotted lines show the overall performance average across lists for the male talker, the female talker, and across talkers, respectively.

Speech perception results for all 20 lists of nonsense BEL sentences, as recorded by an older female talker and a younger male talker, are shown in Figure 2. Because of the dichotomous nature of the response variable, the same generalized linear mixed-effects model used to analyze results from the fill-in-the-blank experiment was also used to analyze the intelligibility results. Fixed effects included talker, list, and the interaction between the two, while random effects included an intercept for each participant. An analysis of deviance revealed a significant effect of talker, χ^2^(1) = 44.7, *p* < .001; a significant effect of list, χ^2^(19) = 343.1, *p* < .001; and a significant interaction between talker and list, χ^2^(19) = 149.9, *p* < .001. The main effect of talker confirmed the higher scores achieved with the older female talker (*M* = 73%, *SD* = 5.0, range: 54%–89%) than the younger male talker (*M* = 60%, *SD* = 6.2, range: 39%–77%). Post hoc contrast tests also confirmed significant differences in performance across lists for both the older female talker, χ^2^(19) = 343.1, *p* < .001, and the younger male talker, χ^2^(19) = 218.5, *p* < .001.

**Figure 3.**
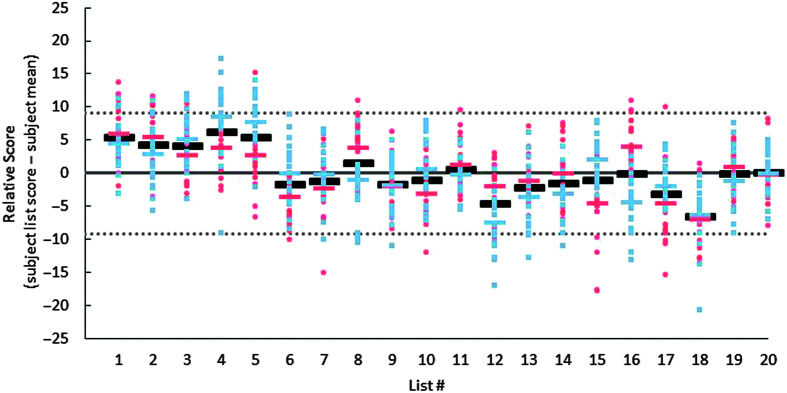
Scores for nonsense Basic English Lexicon sentences recorded by an older female talker (blue) and a young male talker (red), plotted relative to each participant's mean across lists. The red and blue bars indicate mean relative scores for each list for the male and female talkers, respectively, and black bars show averages across the two talkers. The horizontal dotted lines show the 95% confidence interval for performance on all lists across talkers.

To illustrate performance differences between lists, independent of talker differences, scores are replotted in Figure 3, relative to each participant's mean score across sentence lists, with the horizontal bars representing the percentage point deviation from the mean for each talker (blue or red for female or male, respectively) or the mean across talkers (black). As shown in Figure 3, some lists produced consistently better-than-average (e.g., List 1) or worse-than-average (e.g., List 18) performance for both talkers, whereas others (e.g., List 16) produced different results depending on the talker. When averaged across talkers, no sentence list produced performance that was more than 7 percentage points away from the mean, and no sentence list was outside the 95% confidence intervals. The only lists for which mean performance deviated from overall average performance by more than 5 percentage points for both talkers were Lists 1 and 18. Interestingly, in a validation study of the original BEL sentences (Rimikis et al., 2013), performance for nonnative English speakers was also better than average for List 1 and poorest for List 18. Therefore, the differences observed in nonsense Lists 1 and 18 may be due to differences in vocabulary specific to these lists, rather than any effects of word scrambling. Figure 3, and the full dataset provided via the DRUM link (https://doi.org/10.13020/tdf8-6p56), can also be used to select subsets of lists that are more closely equated for performance for a given talker, or across talkers. For example, Lists 1–5 are very well matched, as are Lists 6–20, when excluding Lists 12 and 18.

## Conclusions

A corpus of 500 syntactically correct but semantically incongruent nonsense sentences, matched for vocabulary, number of keywords, sentence length, and talker to the existing BEL corpus, was developed and validated for list equivalency in young normal-hearing listeners. In conjunction with the original BEL corpus, this new nonsense BEL corpus can be used in research related to the use of semantic context in hearing-impaired and other populations and its association with speech understanding and listening effort. With further validation in listeners with hearing loss or CIs, the lists could also be used for clinical testing. The text of the original and nonsense BEL sentences , along with the recordings from all four talkers, are available for download at https://doi.org/10.13020/tdf8-6p56.

## Supplementary Material

10.1044/2020_JSLHR-20-00174SMS1 Supplemental Material S1 Datasheet overview.

10.1044/2020_JSLHR-20-00174SMS2 Supplemental Material S2 Datasheet overview.
