# Supplementary material for: Development and Validation of Sentences Without Semantic Context to Complement the Basic English Lexicon Sentences
Source: J Speech Lang Hear Res. 2020 Oct 13;63(11):3847–54. doi: 10.1044/2020_JSLHR-20-00174 (PMC8582750; doi:10.1044/2020_JSLHR-20-00174)
Supplement: Supplemental Material S1 [file JSLHR-63-3847-s001.pdf]

Supplemental Material S1. Complete list of all 500 nonsense sentences.

List 1

1. the FATHER SELLS with BRIGHT MUSHROOMS
2. the CITY HOSPITAL PLAYS TODAY
3. the HOLIDAY PARK RESTS EASY
4. the MILK is SWEET and VERY STRONG
5. the ELEVEN MEN GROW many TOYS
6. the BEER LOVES OLD CAKE
7. the CHOCOLATE GARDEN OPENS OFTEN
8. my HAT DRINKS in the CROWDED SCHOOL
9. he WEARS his HORRIBLE CHEESE HOME
10. the AMAZING KID is COLORFUL and WEAK
11. a DANGEROUS WEEKEND is REALLY THIRSTY
12. the KIDS TASTE the PARTY SALT
13. these ANNOYING MONTHS BAKED in a GAME
14. that DOCTOR HATES MORE CANDY
15. the BROWN BAR LOST QUESTIONS
16. the GIRL ATE BUSY DRESSES
17. the SISTER and JUICE SMELLED LAZY
18. my GRANDMOTHER WORKS the RED BATH
19. the BEAUTIFUL GROUND ENJOYED the WORKER
20. his VEGETABLES CARRIED in LARGE EGGS
21. the FAST PARADE was BARELY ALIVE
22. our TRAIN MISSED TAKING the CHEESE
23. the GRAY SUN ASKS a STUDENT
24. the CAT WARMS BROTHER on the PLANT
25. my WHITE MOUSE NEEDS my PERFORMER

List 2

1. the PEOPLE WRITE AFTER her SALAD
2. the CLASS BROKE SCARY TWINS
3. the UNIVERSITY SOUNDED their FIVE PLANE
4. the GLASS NOISE OWNED FAR away
5. that LOUD KITCHEN LEARNED to the DOG
6. our PLACES TASTE the CHEAP BABY
7. the SERIOUS LIGHT WORKS for DRINK
8. some GRANDPARENTS LAND LARGE LUNCH
9. the LATE COUPLE MET FRUIT
10. the TEENAGER FORGOT WITH MONKEY
11. the LOST FATHER is REALLY SIMPLE
12. the LADY SEEMS to bring COFFEE SOMETIMES
13. a SHY COUSIN TRAVELED the PEOPLE
14. a CHILD CHASED DOWN the OFFICE

Supplemental material, O'Neill et al., "Development and Validation of Sentences Without Semantic Context to Complement the Basic English Lexicon Sentences," *JSLHR*, https://doi.org/10.1044/2020_JSLHR-20-00174

15. the THIRSTY DISH was EXCITED and BLACK
16. the HUNGRY STREET UPSET the TOURIST
17. her NOISY ACTOR KISSED GRANDPARENTS
18. the MINUTES and FRUIT SING AGAIN
19. our CRUEL COUGH WALKED HORRIBLE
20. her NEIGHBORS are BRIGHT AND NOT SILVER
21. the TEN PROFESSORS will AVOID in DINNER
22. the PRIVATE STUDENTS were FRESH YOUNG
23. the WOMAN was WEIRD in MANY PROBLEMS
24. my PLAYERS LIVE in the FAVORITE CAR
25. the BEARS EAT BROWN PERFORMER

List 3

1. the TEENAGERS TOOK HUNGRY SNACKS
2. my QUEEN BURNED SONGS for the MOUSE
3. the SHORT HORSE EXPLORED BRIGHTLY
4. a THEATER PLANNED FOREIGN BASEBALL
5. the RED LUNCH MADE the FAMILY
6. a KING and CHEF PLAYED the BAND
7. the MANY JOBS EAT WONDERFUL
8. the BLUE BUSINESS BROKE CLOTHES
9. the EVENING and TREE ATE the WINDOW
10. the DARK PUPPY is NEW and ORANGE
11. a CHRISTMAS NURSE is QUITE STRONG
12. the USEFUL GRANDPA SELLS in the FIELD
13. the CATS SCARED FIRE in my PATH
14. the POPULAR RABBIT was LATE this PARTY
15. the ARMY CREATED BIG NIGHTS
16. a FRESH SKY JUMPED HIGH
17. a BIRD ROASTS the FULL CONCERT
18. an EXCITED BICYCLE LOVES BREAKS
19. the RAIN WORKS in the OPERA RESTAURANT
20. the GLASS WAITER CHASES the MOTHER
21. her PORK WON WILD WINE
22. the STORE MEANS VISITORS in the PARK
23. the COMPUTER SINGS NERVOUS CHILDREN
24. the CHEAP BEAR went DOWN that BATTLE
25. this TOURIST LOOKS EXPENSIVE MORNING

List 4

1. the WINDOW EATS in WILD GRASS
2. the FEAR FOUND his TWELVE CHILDREN
3. the HOMEMADE NIGHT WORKED FIRST

4. that DAUGHTER LIVES for the COLD HOME
5. the WEEKEND is EXPENSIVE and NOT SAD
6. the TERRIBLE ACTOR HUGS my CUSTOMER
7. a RUDE GIRLFRIEND GREW in the NEWSPAPER
8. the SIX PARENTS GROW WINE
9. the MUSICIAN FAILED GRAY TREES
10. his STUPID GUEST was POOR and JUICY
11. the STUDENT and APARTMENT BRINGS the BIRD
12. the HOT HORSE is TOO WORRIED
13. the FOOD KNOWS the WONDERFUL HUSBAND
14. an ATTRACTIVE WORM DRINKS by the CHEF
15. the WELCOME BASEBALL eats PERFECTLY SMART
16. my MEAL UPSET DARK PLANT
17. the KITCHEN ADULT ENJOYED OUTSIDE
18. the FAMILY COOKS EVERY CAKE
19. the HARD TEA RAN many DAYS
20. the LAZY FATHER CUT RICE
21. a WIFE COMES SWEET APPLES
22. the TOMATO GARBAGE ONLY was BEER
23. the SONGS SLEEP the TEST and JOKE
24. the TALENTED ANIMALS were HIDING GRANDMOTHER
25. the ROUND PRESIDENT SMELLED the FOREST

List 5

1. our BOY SMELLED AROUND the COOKIES
2. the SALTY NEPHEW SEEMED BAD
3. her CHEF WORKS HAPPY NIGHT
4. the BUSY COFFEE BURNED EARLY
5. the ENGLISH DENTIST RULED the BABY
6. the COUNTRY COUSIN ANSWERS to my BED
7. the PROFESSOR WENT HUNGRY and LONG
8. the NEW STUDENT WALKED EVERYONE
9. the POPULAR FISH is OFTEN SIMPLE
10. a BREAKFAST SINGS in the AFTERNOON ALONE
11. the YELLOW QUESTION is DELICIOUS and DIRTY
12. a FULL NEIGHBOR SWAM in SONGS
13. the TIRED DAUGHTER was VERY TINY
14. the LAKE MADE the HOT FLY
15. her IMPORTANT FARMER has SICK CANDY
16. our DAY ASKED SHORT QUESTIONS
17. the SWEET SLEEP ENDED MARRIAGE
18. the ASSIGNMENT SETS the MANY JOBS
19. the TEA STARTS from the FOOTBALL FOREST

20. a LATE CAT BOTHERED the HOUSE
21. the QUEEN ENJOYS SLOWLY in the MOUSE
22. the MOTHER PREPARES SCHOOL at the KITCHEN
23. their SANDWICH BAKES GAME TOMORROW
24. the MAN CHASED CORN in CLUB
25. my GIRL RAN EVERY SUN

List 6

1. this TEENAGER and BOAT CLIMBED GREAT
2. the SKY DAMAGED EASY WIND
3. the MATH PLANTS SLEPT MEAN
4. the SPICY BUS SWIMS TREES
5. the OLD ADULTS DANCE the BASEBALL
6. the MAN BAKED the MANY STARS
7. that CAT was TROPICAL and ALWAYS RED
8. the NIGHT CARROTS RAN VACATION
9. the CUTE AUNT was my COLD PUPPY
10. the FAVORITE DISH is HEAVY and DIFFERENT
11. the OFFICE TOOK STRANGE WRITER
12. the HOT PICTURES were USUALLY TINY
13. the BEAUTIFUL TREE PLAYS on the AWARD
14. she FELT the GRANDPARENTS ACROSS the OVEN
15. an ANIMAL LIT COMFORTABLE SPORTS
16. the TALENTED RESTAURANT RECEIVED in the PIG
17. the GIRL FLEW the KIND STREET
18. the BLACK BEACHES are FAT to LOOK
19. the STRANGERS PLAY on the BLUEBERRY WATER
20. the PIE HELPS DOWN a WINE
21. the WARM TEAMS FINISH the FLOOR
22. the INSTRUMENTS DROVE to LIFTED in FIELD
23. the CROWDED MUSICIAN SELLS the TEST
24. the HOME LEARNED the QUIET BOX
25. her SCHOOL SCARED SUNSHINE in BABY

List 7

1. the DRIVER EARNED FROM the EGG
2. her CALM CHILD RAN CHEF
3. her LIVING SINGER SLEEPS CLEAN
4. the DRUMS APPRECIATED the GREAT MORNING
5. the BRAVE BOYFRIEND MADE the DOG
6. a PEACEFUL YEAR is EXCITING to VISIT
7. an ACTOR is THOUGHTFUL and VERY LAST
8. the FUNNY PETS COST NOODLES

9. his BOOK TASTES LAZY BUTTER
10. the FOREIGN DANCING was BLACK and FRESH
11. the WATER SENT INTERESTING FANS
12. this PROUD SWEATER was ALWAYS READ
13. the STARVING TV SMELLED on their PEOPLE
14. the CLEAR MONEY was COOKED with NEWS
15. that GROUP is for ROOM EVERY NOVEL
16. a COMMUNITY ATE STRONG STORIES
17. the STRANGER CHEERED the MAN and FIGHTER
18. the SAD PROFESSOR ENJOYED a SAUCE
19. the DOLLARS SIT in a CHURCH DANCER
20. the BORING PARENTS have TEN FLOWERS
21. the CROWD and MEAT TELL the COUNTRY
22. the LARGE FOOD INSPIRED YESTERDAY
23. the TEAM LOOKED KIND in the MEAL
24. the LOVE JOINED the FRIED NEIGHBOR
25. the BEACHES NEED WORD OFTEN

List 8

1. a MAN SHINED AROUND the GAME
2. the CROWDED JUNE was NEVER BEAUTIFUL
3. the STEAMED DOOR LOOKS PRIVATE
4. the SONG TASTED SCARED COUPLES
5. the BLUE JUICE LOSES my FIGHT
6. her NIGHT was NEAR the NEW BROTHER
7. the ENTIRE DOG BOUGHT SOUP
8. the CHICKEN WON our WOODEN WORKERS
9. the BIRTHDAY TOY had our OLD PRIZE
10. the PRETTY LADY is HARD and DANGEROUS
11. the DUCK KEY was STRANGE in NEED
12. they ENTERTAIN TWO DEEP CARS
13. a KIND CABBAGE SERVES to the MORNING
14. the HOUSE EXPLANATION is OFTEN DARK
15. a SIMPLE BEACH is the YOUNGEST SISTER
16. the FRIENDS CLOSE SAD TV
17. they FOUND the GRAPE SCHOOL BRIGHTLY
18. my GRANDFATHER SINGS EVERY APARTMENT
19. that TERRIBLE MAID WATCHES GIFT
20. his FLOOR was SERIOUS and REALLY HAPPY
21. the OCEAN SPILLED QUICKLY on EDUCATION
22. the RESTAURANT SLEEPS the THREE HOLIDAYS
23. the BEST GIRL LOST the SUN
24. its HOPE PRACTICES until LATE PET

Supplemental material, O'Neill et al., "Development and Validation of Sentences Without Semantic Context to Complement the Basic English Lexicon Sentences," *JSLHR*, https://doi.org/10.1044/2020_JSLHR-20-00174

25. the CLOSEST TEAM ATE BETTER

List 9

1. the FRIEND DESTROYED about COLORFUL HOUSES
2. the BEST BUS is SOFTLY SMALL
3. her ADULT STUDIED EARLY RAIN
4. his CHINESE PLANTS TELL LAZY
5. the BLACK UNCLE CALLED her CROWD
6. she DANCED a VERY LONELY CAR
7. their SAD POTATOES ATTRACT VACATION
8. the TINY CUSTOMERS FIRED for the HOURS
9. the CHEAP WORKER is BAD but RICH
10. her WAITER TASTED YESTERDAY on his ANIMAL
11. the SONG LOVES in RELAXING BOY
12. the BEAR PLAYED some DANGEROUS DRINKS
13. the GIRLFRIEND OWNS the AUDIENCE and BOSS
14. a FAR HAT BOUGHT CHILDREN
15. the BIG SON DROVE many STORIES
16. the LADY CRIED the FAMOUS CLASS
17. the TRIP TRAVELED SOME ROOM
18. the YOUNGEST DANCE SEEMED SCARY
19. the INTERESTING FOOD is STRAIGHT WELL
20. the FRUIT LEARNED FIVE WRITERS
21. the CUTE PROFESSOR was CITY and SUMMER
22. the PERFORMER WATCHED PRETTY TALENTED
23. the LAWYER ENJOYED USUALLY TOGETHER
24. that EARTH CHILD WENT SPOILED
25. she HATES the SCIENCE FAST and ALWAYS

List 10

1. the BIG STRANGER INTERESTED his INSTRUCTOR
2. the COURSES OFFER THIRSTY FISH
3. the BEST PASTA DRANK NOISY
4. the GRANDMA SELLS DELICIOUS COLLEGES
5. her BIRD and GIRL READ the BEAR
6. the LAST GRANDPA ATTRACTS the EGGS
7. the CAMPERS SEEMED EMPTY and SOFT
8. the THREE BRUSHES SHARED NEWSPAPER
9. the RUDE student SWIMS the YOUNG FRIEND
10. my WHITE EXAM was CHRISTMAS and LONELY
11. the FINAL CHEF was SCARED too BAD
12. the SISTERS PLAYED THIN MARKET
13. the TIRED STATION COOKS for her CLOTHES

14. the PAINTER CUT MILK EVERY day
15. the BORING GRANDMOTHER LAYS UNHAPPY
16. the MEAN ROOM CRIED BOYFRIEND
17. the KIDS VISITED the ANGRY ATTENTION
18. the CLASS TOOK ART FOOD
19. these BOOKS FELT with my TINY SON
20. the BOILED GROUP USED in the CAT
21. her MOTHER SMELLS QUICKLY to the TEACHER
22. the MANY TRAVELERS GAVE ONION
23. my BAGS HELPED the LAKE EARLY
24. the NICE SHOW is NEVER TROUBLED
25. the CHILD ARRIVED MUSEUM at her DUCK

List 11

1. the WINDOWS LEARNED in BROWN SECRETARY
2. the MEAL PLANNED TWENTY KIDS
3. that COOL ROOM DRINKS HERE
4. a DANGEROUS BIRD was REALLY ORANGE
5. the TWO GLOVES had their ENGLISH HORSE
6. the CHICKEN MOVIE CLIMBED the SON
7. the GROCERY PERSON NEEDS FARM
8. the CAKE BIT in the BETTER SOUP
9. the DIFFICULT JUICE was the BIRTHDAY TEST
10. the TASTY NIGHT was THREE and DARK
11. the TROUBLED GRADE is SENT EASILY
12. a RADIO FEELS PROFESSOR LOUDLY
13. the SMALL TEA SELLS over an APARTMENT
14. our HOMEWORK BUYS MORE SKY
15. our RABBIT STARTED SICK CUSTOMERS
16. a COMFORTABLE BOYFRIEND COVERED the COUSINS
17. the SNAKE PLAYED the UNFAIR WEDDING
18. they HATE GREEN MINUTES on the CARD
19. the MATH STORE was ALWAYS RELAXING
20. the SISTER and CLOUD SCREAMED their MOTHER
21. the MEETING LIVES LATE on the FENCE
22. the WHITE MONEY GAVE KITTEN
23. his SNOWMAN STOLE SPANISH DAILY
24. the LITTLE GIRLFRIEND STARTS in that MUSIC
25. the PARK did FAST BLACK FOOD

List 12

1. a WIND CAME DOWN the GIFTS
2. the SCHOOL BAG had the DRY BROTHER
3. the NICE GOAT DRIED GREAT
4. this DINNER FAILED the CITY STORY
5. the TERRIBLE CUSTOMER ROLLED every MOTHER
6. the POTATOES TOOK CUTE CARROTS
7. the MANY CHILDREN RECEIVED KITCHEN
8. her DOCTOR and AIRPLANE PICKS BALL
9. the HAPPY STREETS HELPED QUIETLY
10. the BEDROOM LEAVES CHEWED the SISTER
11. a LARGE HAIR was HONEST and UPSET
12. my YOUNGEST FARMER was ALWAYS CURLY
13. her LOVING FLOWERS LAUGH in a MAP
14. the YEAR LOVES for those STRONG RATS
15. the GIRL TASTES SICK SON
16. the BUSY STAIN WAITS BUSINESS
17. they WASHED and BOUGHT my LOUD HILL
18. the RUG GROWS the FAT PATIENT
19. the PRETTY SKY is ANGRY and EXPENSIVE
20. the YELLOW PLANTS COOLED in the SHOPPER
21. her ROADS BOILED QUICKLY at the PICTURE
22. she RAN the INTERNATIONAL UNCLE the CARROTS
23. the CHEF SHOWS DARK BUNNY
24. the ANSWER FLEW in the BLUE ANIMALS
25. the AIR EATS THINGS through the SHOES

List 13

1. another PASTA MADE AROUND a DATE
2. her VEGETABLES PRACTICED HIGH GARBAGE
3. their FUN TRUTH LISTENS FIRST
4. the NIGHT ENDED the TRAIN FRIEND
5. her PERSON LOOKED FAVORITE and BAD
6. the UNDERGROUND CLOTHES FLY the PARTY
7. their PANTS WATCHED BOSS with the DOOR
8. the FRIENDLY CHILDREN BOILED CAMPUS
9. the RUINED and SAD MEAL is DIFFICULT
10. her HOT MUSIC is THREE and WOODEN
11. the VOICE ATTRACTS on the ROMANTIC KEY
12. a METAL PEPPER was USUALLY SPORTS
13. the STATION TELLS to TERRIBLE HOURS
14. this GREEN GIRL VISITED at NOISE

15. a DOG LASTED OLD MOVIES
16. our CLASSICAL FLIES WERE LONELY
17. the FLAGS LOVE the LOST TEAM
18. the TENNIS MACHINE was BROWN and UNHAPPY
19. the FAMILY ASSIGNED for the LOUD CHAIR
20. those STUDENTS OPENED DIRTY and MANY
21. the SHOW NEEDS EARLY TODAY
22. a WHITE PROJECT SOUNDS the PETS
23. the ARTIST WALKED in GOOD BOYFRIEND
24. the KITTEN SAT STORES and WATER
25. the MUSEUMS SELL PROUD SALT

List 14

1. the CHICKEN STUDIES ABOVE the JOKES
2. they WROTE CAKE in the GREEN POLICE
3. the CLUB RABBITS LOOK FRIED
4. her DOG SERVES me the PEACEFUL GRANDPARENTS
5. the HEALTHY STREET is FOREIGN SISTERS
6. the HOME DREAMED in the CLEAN CLOTHING
7. the FOUR PARENTS BAKED BOSS
8. the PERSON STOPPED SLOW DAUGHTER
9. a NEW THIN DOOR is YOUNGEST
10. my SMALL LIFE was FISH and LITTLE
11. the FAMILY CHASED BIRTHDAY NOVEL
12. the HAPPY STORE is SUDDENLY LONG
13. the COUPLE BOUGHT BOOK and STUDENT
14. the SAD TREE ATE from my MAN
15. the AUTHOR PLAYED QUIET GIRL
16. a LONELY HOUSE had ANGRY YESTERDAY
17. a ROOM WATCHED the TIRED RESTAURANT
18. the TWIN VISITORS TRUST at the LADY
19. the LARGE SPORTS SOLD MONKEY
20. their PROBLEMS and DINNER HUNG the DRINKS
21. that CHILD LIVES her MEMBERS TODAY
22. a HELPFUL MOVIE EXPECTED the PLANTS
23. the PICTURE TELLS the MANY COUSIN
24. the HORRIBLE COMPUTER was BUSY and TALL
25. the MARKET SWINGS REALLY of the DRIVER

List 15

1. the WEEK FELL DURING their ACTOR
2. the BIRTHDAY DESSERT SHINES the SOUP
3. the SALTY MOUSE CARRIED WARM

4. they GAVE the ITALIAN THEATER and PROJECT
5. a FOOTBALL LUNCH CHASED the SHIRT
6. the KIDS ROASTED FOUR FANS
7. the FAST HELP LIKES AUNT
8. that LAWYER ENDED SMALL DOGS
9. the STRESSFUL CLOTHES are the FRENCH BAR
10. the NICE TABLE is VEGETABLE and GIANT
11. the FOOD CELEBRATED on the CHEAP PAN
12. the FAVORITE HUSBAND was REALLY FARM
13. the LITTLE BREAK BUYS off the GIFTS
14. a BROWN HOMEWORK was SMOKED in BOY
15. the OLD BALL was SOFT and POPULAR
16. their CHILD SANG GREEN WAITRESS
17. the TOWN WATCHED her PREGNANT BAGS
18. the ANGRY CHOCOLATE COMPLETED at the KITTEN
19. those HOLES VISITED LOUDLY in a FAMILY
20. the TIRED HOLIDAY LOOKED in WOMAN
21. that FOREST NEEDS RESTAURANT TODAY
22. the HEALTHY TIME LOVED VACATION
23. they DIG SIX GAMES on the SUN
24. the UNHAPPY MEAT was LONG DAILY
25. the TEACHER WENT DIRTY FRIENDS

List 16

1. the PICTURE RECEIVED for YELLOW OCEAN
2. our PRIEST SCREAMED TROPICAL BUSINESS
3. the USEFUL LUNCH DESTROYED FRIENDLY
4. the MANY ROOM ATE around the AUNT
5. the QUIET WAITER CAME the TEAM
6. the PEPPERS CHEERED WORLD PERFECTLY
7. the RELIGIOUS WEATHER GREW ARTIST
8. the RATS SEEMED the TEN COUSINS
9. the FOUR SONGS are SICK PLATES
10. he TOOK VERY in the UGLY GOD
11. the BEAUTIFUL PATIENT was COLORFUL and STORMY
12. the TOY FOREST was QUICKLY BAD
13. the SMALL HOME LOVES in my FISH
14. the CARS WORKED HOT CLASS
15. a CALM PIE NEEDS for FLOWERS
16. the NEWS BELIEVES STILL BANANAS
17. the MATH SON BAKES SUDDENLY
18. the BIG CHILDREN were EXCITED and GLASS
19. the DISH TRAVELED CLEAN MONEY

20. the CATHOLIC KITCHEN BROKE many SISTERS
21. her SUPPORT has WARM and WEEKLY TALKING
22. the GARDENER LOOKED ALWAYS CROWDED
23. the LITTLE TREES had CHERRY COUPLE
24. their PERFORMER SANG ADULTS LOUDLY
25. the SWEET MONKEY WANTS GLASSES

List 17

1. the HAT HEARD ACROSS the VEGETABLES
2. the CLASS FELL DEDICATED GARDEN
3. the HOT HOLE SPEAKS MORE
4. the SUPERMARKET GAVE UGLY STAGE
5. the TALENTED SALAD SAT the COUPLE
6. the CHICKEN PRESIDENT is BROKEN and DIFFERENT
7. the TEN NURSES CAME up the MOVIE
8. the LAZY MOUNTAIN READS LANGUAGES
9. a DIVORCED PINK SPEECH was HUGE
10. the FAT FRIENDS were DIFFICULT and FUNNY
11. the SANDWICH DREW the TWO SCHOOLS
12. the SWEET GROUP is VERY SLOW
13. a STRANGE FATHER SEEMED with the CHEESE
14. the LESSON SAW at the MANY PATIENTS
15. the PICTURE GREW BLUE MUSIC
16. the TELEVISION TABLE TASTED TOMORROW
17. she WARMED ENGLISH POPULAR BOOKS
18. the CARS HELP FOREIGN ACTOR
19. the ARTIST HIKED in the DEEP DAUGHTER
20. the GIFTS and COMPANY FOUND that POOL
21. the SMART BREAD OPENS the PIG
22. the TASTY TEACHERS NEED in WOMAN
23. the YOUNG SUN is COMPLETELY GREEN
24. the SHORT GOAT BUYS SHOW
25. the MOUSE WALKED TOO BAKED

List 18

1. the ONLY ARM is LOUD and FAR
2. the WAITERS LASTED TWO KEYS
3. the ITALIAN MEAL ASKS RIGHT
4. my LIGHTNING TRAVELED on our THIRTY CLUBS
5. our ENGLISH MEAT ASSIGNS many CATS
6. the HELP PRACTICES BROKEN CORN
7. the PINK LEG was ALWAYS OLD
8. the GREAT LADY SAVES BAND

9. our FAITHFUL APPLE and BREAKFAST were HAPPY
10. the BOX ENDED the CROWDED FARMER
11. the MANY BOOKS never JOINED DOCTOR
12. the ORANGE CITY WROTE in the GAME
13. a WARM DAUGHTER WON for DESSERT
14. a PLAYER MADE KIND BUTCHER
15. the FAR SHIRT FELL GARAGE
16. the HOMEWORK SERVED the AMAZING AUTHOR
17. her SMALL FLOOR CUT the NOISE
18. the VISITOR DISAPPEARED for the BUSY PROFESSOR
19. the RAIN was BEST and TOO CURIOUS
20. the PERFECT SOCCER PHONE is the BUTTONS
21. the COFFEE WEEKS TASTED AWARD
22. the LIVES DRINK in SICK MILK
23. the EXPENSIVE HUSBAND GIVES the MARKET
24. the HOTEL SELLS to the WOOD ADVICE
25. a HARDWORKING CAKE had no SPICY NEIGHBOR

List 19

1. the INTERESTING CUP was SMALL and FRIENDLY
2. the SEA EATS THEIR ARTIST
3. the PLASTIC BIRD LISTENED FIRST
4. the GIFT FLEW every SHY BED
5. the QUIET BOAT MADE PEACEFULLY
6. the GOLD QUESTION HUGS our GAME
7. a BUSINESS FIT WELL for the FRENCH
8. the JAZZ CHILDREN TELL CARROTS
9. the TALL PET and APPLE are NEW
10. the FUN COUPLE were CHEAP and BORED
11. her STORIES WORK PRETTY PUPPY
12. the EXPENSIVE STUDENT is TOO DRY
13. the GREAT PHONE went DOWN the CHAIRS
14. the SAME FAMILY LOST to FINGER
15. my DAY LOOKED RED NURSES
16. the DAILY CONFUSING RING is not HUNGRY
17. my JEANS WALKED the OFFICE NEIGHBORS
18. the BLUE RABBIT SANG TWINS
19. their SINGER RANG on the DIFFICULT GRANDFATHER
20. the RICE and EVERYONE PAID LIGHT
21. a SONG SLEPT AWAY the WOMAN
22. that WOODEN BROTHER RECEIVED HARD
23. the BABY STUDIES OVER the RIVER
24. the PLATE JOINED the MUSIC OFTEN

25. the NEWSPAPER SOUNDED BEANS and ITALIAN

List 20

1. my DENTIST BROKE ABOUT the CHILDREN
2. that FOREST KNOWS the RED GOALS
3. the FIVE GRANDPARENTS BOUGHT the MUSIC
4. a LEFT PIG was BIG and SHY
5. the RICH ARTIST HURT FALL
6. our AUNT DREW the WAR SOON
7. the LITTLE PEOPLE RAISED new BALL
8. the SOCCER HAND was LONG and CRAZY
9. the TOYS CELEBRATED SCARED RABBIT
10. the SOFT COLOR is TOO FUN
11. the THIRSTY DAY CHOOSES in the WORKER
12. the HOLE SPEAKS his OLDEST GUEST
13. my GRAPES are YELLOW and QUITE DIFFICULT
14. the TEACHER PLEASED LAKE in the MOUSE
15. the WATER CUT through the HELPFUL NEWS
16. the LEAVES END TOOLS TOGETHER
17. he PLAYS the SPEECH WITH the HOLIDAY
18. the LAST PLAYER DRANK EVERYONE
19. the DEEP PAPER STAYED QUIETLY
20. my UNCLE KICKED HORRIBLE RULES
21. the BORING KNIFE UPSET their STEAK
22. his MEAN DAUGHTER CLEANED on FAMILIES
23. the TASTY TEAM will CHANGE EASILY
24. a FUNNY HOUSE RUNS with the MOVIE
25. the COLD NANNY SCORED the QUESTIONS
