# Supplementary material for: Development and Validation of Sentences Without Semantic Context to Complement the Basic English Lexicon Sentences
Source: J Speech Lang Hear Res. 2020 Oct 13;63(11):3847–54. doi: 10.1044/2020_JSLHR-20-00174 (PMC8582750; doi:10.1044/2020_JSLHR-20-00174)
Supplement: Supplemental Material S2 [file JSLHR-63-3847-s002.pdf]

Supplemental Material S2. List of the original words along with the words that were accepted as having a similar semantic meaning.

Context Sentences	Key	Participant responses (Synonym)
The milk and cheese smelled __________.	horrible	stinky
My strong __________ carried my brother.	father	dad
The family ___________ in an expensive restaurant.	ate	dined
This new computer is ____________ useful.	quite	very
The ___________ broke a glass window.	baseball	ball
My grandmother __________ a chocolate cake.	baked	made
The party game was _________ easy.	really	pretty
That __________ sells cheap clothes.	store	shop
The kitchen garbage smelled ____________.	terrible	horrible, bad
The ____________ write simple problems.	professor	teacher
The trees ____________ sweet apples.	grow	produced (2)
Some ____________ drink black coffee.	people	folks
Her grandparents are serious and sometimes ____________.	cruel	stern
The last year was __________ and peaceful.	calm	quiet, relaxing
The popular club is often __________.	full	crowded
My neighbor sings country __________.	songs	music
The tiny fly __________ everyone.	bothered	annoys
The fish swam __________ in the lake.	slowly	quickly
A __________ country is exciting to visit.	foreign	new
The church __________ inspired the community.	group	congregation
The news was on TV __________ morning.	every	each
The wind damaged the __________ boat.	tiny	little
The stars __________ the night sky.	lit	illuminated
Her apartment was near the private __________.	school	campus
The __________ and trees look beautiful.	plants	flowers
The restaurant __________ red wine.	sells	served, serves
She drove the bus down the __________.	street	road
The spicy carrots were her favorite __________.	dish	food
The strange animal scared the __________.	baby	kid
My brother sleeps until __________ morning.	late	mid
The small boy seemed __________.	sad	scared (2)
Our __________ drinks orange juice.	mother	mom
A __________ kitten climbed over the fence.	little	young
The chicken soup was a __________ meal.	tasty	good
That __________ animal is cute but dangerous.	tiny	small
A summer vacation is __________ relaxing.	always	very, quite
The __________ learned about earth science.	class	students (2)
The boss __________ the lazy waiter.	fired	scolds
Some writers __________ interesting stories.	tell	write
They played fast __________ on the radio.	music	songs
The best worker went on the __________.	trip	vacation
Their __________ son danced well.	famous	talented

The __________ destroyed some plants.	rain	storm
Those cute animals __________ the plants.	chewed	eat (2)
His grandma and grandpa helped the __________.	kids	child
The big __________ felt empty.	room	house
The honest mother is loving and __________.	nice	caring
The lonely ducks swims in the __________.	lake	pond (2)
Her uncle __________ quietly for the answer.	waits	listens
The instructor __________ books to her class.	gave	brought, brings
These colleges offer many __________.	courses	classes
The __________ used soft brushes.	painter	artist
The Christmas __________ interested my son.	show	play
The __________ lays tiny eggs.	bird	chicken
The chef __________ pasta every day.	cooks	made
Her youngest son was always __________.	upset	crying
They __________ salty meat in the pan.	roasted	cooked (2)
That __________ carried six bags.	boy	man (2)
A lonely person is usually __________.	unhappy	sad
They loved the French __________ and dessert.	food	meal, dinner
The party __________ for three hours.	lasted	continued
The teacher __________ homework daily.	gave	assigned (2)
The old shirt was warm and __________.	soft	fuzzy, comfortable
That fast __________ chased a mouse.	kitten	cat (2)
The student studies in the quiet __________.	room	library
The __________ serves dinner and drinks.	restaurant	diner
My cousin __________ my a birthday cake.	baked	made
The tropical __________ had many trees.	forest	rainforest
He __________ loudly in the crowded room.	screamed	yelled
The green __________ look healthy.	plants	grass
The artist took a __________ picture.	beautiful	pretty
The performer worked for little __________.	money	pay
The __________ looked perfectly calm.	ocean	sea
The boss tells horrible __________.	jokes	stories (2)
The Catholic priest sang __________.	songs	hymns
The slow computer had many __________.	problems	viruses
That new book is __________ helpful.	really	very
The __________ hung above the door.	picture	sign
That goat __________ in the deep hole.	fell	jumped (2)
The talented artist drew a __________.	picture	portrait
The jazz __________ sounded great.	singer	concert, ensemble
The lesson seemed too __________.	difficult	complicated
The bird flew __________ the sea.	over	above
The company __________ foreign cars.	buys	purchased
The young woman is __________ smart.	very	incredibly

A pretty boat went down the __________.	river	stream
The two friend __________ up the mountain.	hiked	climbed
The nurses work hard every __________.	day	shift
The nurses work hard every __________.	day	shift
The couple sang the __________ well.	song	anthem
The hot sun __________ the pool.	warmed	heated
The divorced couple sat at the __________.	table	desk
Our business paid for the daily __________.	newspaper	paper
The vegetables grew in the green __________.	garden	yard
The __________ wrote thirty books.	author	writer
The helpful nanny cleaned the __________.	house	room (2)
The __________ lady give advice.	kind	nice
The team __________ goals easily.	scored	shoots
**Context Sentences**	**Key**	**(Same category)**
The _________ loves sweet candy.	girl	child, boy
The park opens in __________ months.	eleven	two
The worried adult ____________ home.	ran	walks, came
The ____________ bears eat fruit.	brown	black (2)
The woman met her favorite ____________.	actor	celebrity
The glass ____________ broke in the kitchen.	dish	cup
The twins live with their ____________.	grandparents	grandmother
The sun sets in the late __________.	afternoon	evening (2)
The hungry __________ made a sandwich.	girl	boy, man
Her black __________ looked funny.	sweater	shirt
His __________ tell boring stories.	parents	grandparents
Her thoughtful __________ sent flowers.	boyfriend	husband (2)
The kind __________ helps strangers.	girl	person, woman
The fat pig __________ on the floor.	slept	lay, laid
The talented __________ received an award.	writer	musician
They bought three __________ cars.	blue	red
The __________ juice spilled on the floor.	grape	cranberry
Our __________ drinks orange juice.	mother	father
The snowman had __________ green gloves.	two	four
The dark cloud covered the __________.	sky	sun
The meeting starts in __________ minutes.	twenty	five, ten
That __________ test was really difficult.	English	math (2)
The three __________ did their math homework.	cousins	kids, siblings
His grandma and grandpa helped the __________.	kids	son
The happy __________ laugh at the story.	children	teenagers, people
They took a __________ picture every year.	school	family (2)
The shopper bought __________ things.	many	ten
My grandmother read the __________ quickly.	newspaper	book (2)
The __________ team practiced at night.	tennis	soccer

The fans watched __________ games.	football	baseball
Her __________ watched movies with another girl.	boyfriend	friend
The artist studies __________ and French.	Italian	spanish
The __________ ring fit her finger.	gold	diamond
The __________ sandwich came with salad.	chicken	turkey
The __________ sandwich came with salad.	chicken	beef
The dedicated __________ help patients.	nurses	doctors, surgeon
My __________ made wooden chairs.	grandfather	father
Their oldest __________ plays with toys.	daughter	child, son
A little __________ runs through the forest.	rabbit	fox
The player __________ the soccer ball.	kicked	hits
Her right arm and __________ were broken.	leg	hip
The hungry teenagers eat __________.	snacks	pizza
**Context Sentences**	**Key**	**(Antonyms)**
The wooden door was hard to __________.	close	open (2)
Our team practices every __________.	night	day
The __________ night was comfortable and relaxing.	cool	warm
The customers __________ black tea.	hate	love, loves
The city bus is usually __________.	early	late
The show __________ early today.	ended	started
The worker hurt his __________ hand.	left	right (2)

**Nonsense Sentences**	**Key**	**(Synonyms)**
The weekend is expensive and __________ sad.	not	never
A child __________ down the office.	chased	ran
The hot horse is __________ worried.	too	very
A dangerous weekend is ____________ thirsty.	really	very
The painter cut milk __________ day.	every	all
The __________ travelled some room.	trip	tour
The math store was __________ relaxing.	always	very
The sweet group is __________ slow.	very	really
**Nonsense Sentences**	**Key**	**(Same category)**
My __________ ran every sun.	girl	son
The __________ and cloud screamed their mother.	sister	child, boy
They wrote cake in the __________ police.	green	blue

Supplemental material, O'Neill et al., "Development and Validation of Sentences Without Semantic Context to Complement the Basic English Lexicon Sentences," *JSLHR*, https://doi.org/10.1044/2020_JSLHR-20-00174
